# Performance of the Lot Quality Assurance Sampling Method Compared to Surveillance for Identifying Inadequately-performing Areas in Matlab, Bangladesh

**Published:** 2007-03

**Authors:** Abbas Bhuiya, S.M.A. Hanifi, Nikhil Roy, P. Kim Streatfield

**Affiliations:** ICDDR,B, GPO Box 128, Dhaka 1000, Bangladesh

**Keywords:** Lot quality assurance sampling, Immunization, Health, Family planning, Comparative studies, Bangladesh

## Abstract

This paper compared the performance of the lot quality assurance sampling (LQAS) method in identifying inadequately-performing health work-areas with that of using health and demographic surveillance system (HDSS) data and examined the feasibility of applying the method by field-level programme supervisors. The study was carried out in Matlab, the field site of ICDDR,B, where a HDSS has been in place for over 30 years. The LQAS method was applied in 57 work-areas of community health workers in ICDDR,B-served areas in Matlab during July-September 2002. The performance of the LQAS method in identifying work-areas with adequate and inadequate coverage of various health services was compared with those of the HDSS. The health service-coverage indicators included coverage of DPT, measles, BCG vaccination, and contraceptive use. It was observed that the difference in the proportion of work-areas identified to be inadequately performing using the LQAS method with less than 30 respondents, and the HDSS was not statistically significant. The consistency between the LQAS method and the HDSS in identifying work-areas was greater for adequately-performing areas than inadequately-performing areas. It was also observed that the field managers could be trained to apply the LQAS method in monitoring their performance in reaching the target population.

## INTRODUCTION

Health-programme personnel always aim to increase the level of use of the services they provide. Despite all-out efforts, the use of services often remains lower than expected and/or the level of use is not same for all areas or for all the service components. Focusing attention on deficient areas can help improve the situation. A pre-requisite for focusing attention is the identification of deficient areas with a known degree of reliability. Data from surveillance and cross-sectional surveys are commonly used for assessing the level of use of services. Surveillance being very resource-intensive is not practical for this purpose. The cross-sectional survey, especially the 30 cluster-sampling scheme with 210 respondents, is considered to be reasonably practical and has been in use for monitoring the coverage of services under the Expanded Programme for Immunization (EPI) for quite some time (1). In fact, the task of covering 210 respondents per estimation area, if one looks carefully, is not so small a task when the performance of areas covered by the lowest-level workers—quite often large in numbers—is to be assessed. In addition, the task of data analysis and their interpretation also become technical, requiring expertise beyond the domain of programme management. In this respect, the lot quality assurance sampling (LQAS) method is simpler, resource- and time-efficient for it is based on a much smaller sample size and easy to apply (2, 3). The LQAS method is also effective in improving public-health services, like immunization coverage, by identifying low-performing areas in some settings (4, 5). Although the LQAS method has been used for identifying inadequately-performing areas in many settings (4) and, in some instances, compared to cross-sectional surveys (6, 3), its performance in the field has not been compared with those based on more rigorous data-collection methods, such as surveillance.

Keeping the above in mind, the present study aimed at comparing the proportion of inadequately-performing areas identified by the LQAS method with those derived using data from the Health and Demographic Surveillance System (HDSS) of ICDDR,B in Matlab. The consistency between the LQAS method and HDSS-based classification of areas in terms of performance was also examined. The paper also documents the experience gained in implementing the tool by the supervisors of the health and family-planning programme at the lowest level of implementation.

## MATERIALS AND METHODS

### Description of LQAS

In the LQAS method, a defective article is defined as one that fails to conform to specifications in one or more quality characteristics. A common procedure in the method is to consider each submitted lot of product separately and to base the decision on acceptance or rejection of the lot on the evidence of one or more samples chosen at random from the lot (7).

Application of the method requires specification of three numbers. One is the number of articles ‘*N*’ in the lot from which the sample is to be drawn. The second is the number of articles ‘*n*’ in the random sample drawn from the lot. The third is the acceptance number ‘*d*’. The acceptance number is the maximum allowable number of defective articles in the sample. More than ‘*d*’ defectives will cause the rejection of the lot. For instance, if we have a situation with *N*=50, *n*=5, and *d*=0, it implies that “Take a random sample of size 5 from a lot of 50. If the sample contains more than 0 defectives, reject the lot; otherwise, accept the lot.” The LQAS method uses the binomial probability to calculate the probability of accepting or rejecting a lot.

To apply the above in the context of delivery of health services, for example, vaccination coverage, let us assume the coverage of DPT1 for a health area as *p*. In a health area with an infinitely large population, the probability *P*(*a*) of selecting a number *a* of vaccinated individuals in a sample size *n* is calculated as:

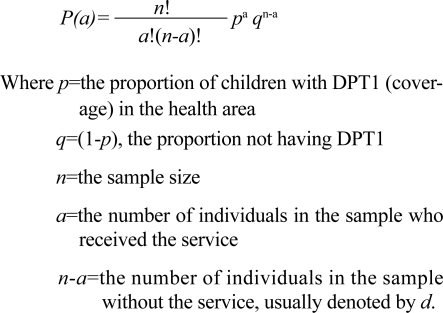


The LQAS method aids programme personnel in choosing the sample size and the permissible value of *n*–*a* and interpreting results. To use the LQAS method in the context of health programmes, for example EPI, the following five initial decisions must be made (8–10): first, the health system manager must select the intervention to assess. In our example, it is the coverage of DPT1; second, select the work-area whose coverage is to be assessed; third, select the target community to receive the intervention (infants in the case of DPT1); fourth, a triage system must be defined for classifying the level of coverage as adequate, somewhat inadequate, and very inadequate. This needs to be decided by programme managers, policy-makers, or other stakeholders of the EPI; and fifth, levels of provider and consumer risks. [Provider risk—probability of wrongly classifying a work-area/provider as very unsatisfactory, which can put the reputation of the worker at risk; Consumer risk—probability of wrongly classifying a very inadequately-performing health area/worker as adequate which can put the inhabitants in the area at health risk]. In most cases, it may be around 10–15%.

Using information from the above five decisions, a series of operating characteristics curve, or their corresponding probability tables can be constructed with the above binomial formula. [An operating characteristics curve depicts the probabilities of accepting a lot based on the proportion of non-conformance in the lot, the sample size, and the value of *d*, allowable non-conformances. An operating characteristics curve enables decision-makers to examine the possible risks involved]. From the operating characteristics curves, one can select the sample size (i.e. *n*) and the number of unimmunized individuals allowed (i.e. *d*) in the LQAS sample for a given level of provider and consumer risk before deciding that a health area has a sub-standard coverage.

Let us assume that a consensus has been reached among various stakeholders of the EPI that EPI centres with 80% or more infants in their catchments area receiving DPT1 can be considered as performing adequately. While the EPI centres with a coverage rate of 50% or less ought to be considered as inadequately performing and be identified for attention, the ones in the mid-range of 50–80% may be considered somewhat adequate, and for the time being no special attention is needed. Using these information, probabilities of detecting ‘adequately-performing’ or ‘inadequately-performing’ EPI centres can be calculated. Table 1 presents such probabilities along with provider and consumer risks for various combinations of sample sizes and maximum allowable unimmunized infants in the sample.

**Table 1.** Example of application of the LQAS method to detect the probability of 80% or 50% coverage of health-area residents with respect to vaccination according to various sample sizes and number of unimmunized children

Sample size (n)	No. in the sample unimmunized (d)	Probability of detecting health areas with 80% coverage as adequate (a)	Probability of detecting health areas with 50% coverage as inadequate (b)	Provider risk (1–a)	Consumer risk (1–b)	Total classification error (1–a)+(1–b)
8	0	0.17	1	0.83	0	0.83
	1	0.50	0.96	0.50	0.04	0.54
	2	0.79	0.83	0.21	0.17	0.38*
	3	0.94	0.64	0.06	0.36	0.42
12	0	0.07	1.00	0.93	0.00	0.93
	1	0.28	1.00	0.72	0.00	0.73
	2	0.56	0.98	0.44	0.02	0.48
	3	0.80	0.93	0.20	0.07	0.28
	4	0.93	0.81	0.07	0.19	0.27*
	5	0.98	0.61	0.02	0.39	0.41
14	0	0.04	1	0.96	0	0.96
	1	0.20	1	0.80	0	0.80
	2	0.45	0.99	0.55	0.01	0.56
	3	0.70	0.97	0.30	0.03	0.33
	4	0.87	0.91	0.13	0.09	0.22*
	5	0.96	0.79	0.04	0.21	0.25
19	0	0.01	1	0.99	0	0.99
	1	0.08	1	0.92	0	0.92
	2	0.24	1	0.76	0	0.76
	3	0.46	1	0.54	0	0.55
	4	0.67	0.99	0.33	0.01	0.34
	5	0.84	0.97	0.16	0.03	0.20
	6	0.93	0.92	0.07	0.08	0.15*
	7	0.98	0.82	0.02	0.18	0.20
28	5	0.50	1	0.50	0	0.50
	6	0.68	1	0.32	0	0.32
	7	0.81	0.99	0.19	0.01	0.20
	8	0.91	0.98	0.09	0.02	0.11
	9	0.96	0.96	0.04	0.04	0.08*
	10	0.99	0.90	0.01	0.10	0.11

*Optimal decision rule for a sample size;

LQAS=Lot quality assurance sampling

Source: Grant EL, Leavenworth RS. 1988:391–425 (7)

The probabilities in Table 1 were calculated using the binomial formula. In each case, the upper and the lower threshold of the triage system were 80% and 50% respectively. To illustrate the calculation, let us take the example of *n*=12 and *d*=3. To calculate the probability of wrongly classifying a work area/provider as inadequate, we first have to calculate the probability of having three or less unimmunized children—of 12 children—in an area with 80% coverage. Finally, we have to subtract this probability from 1 to get the probability of wrongly classifying a work-area/provider as inadequate even if they could well be not inadequate.

The probability of 0 unimmunized (equals to all 12 immunized) children—of 12 children—with 80% coverage is=

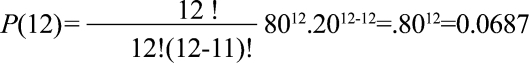


Similarly, probability of 1 unimmunized (equaling 11 immunized)=

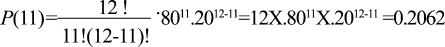


Probability of 2 unimmunized (equaling 10 immunized)=0.2835

Probability of 3 unimmunized (equaling 9 immunized)=0.2363

Therefore, the probability of having three or fewer children unimmunized in an area with 80% coverage is=0.0687+0.2062+0.2835+0.2363=0.7946. This also implies that, with 80% coverage in the area, there is a chance of 0.2054 (1–0.7946) to have three or fewer unimmunized children. Thus, if one, on the basis of having three or more unimmunized children among 12 children, declares that the performance of the area/health provider as inadequate has a chance of misclassifying the area in 20.54% of time. This puts the provider at risk of being wrongly classified as inadequately performing.

On the other hand, with 50% coverage, the probability of having three or fewer children unimmunized is=*P*(12 immunized)+*P*(11 immunized)+*P*(10 immunized)+*P*(9 immunized)=0.0002+0.0029+0.0161+0.0536=0.0729. This implies that, with 50% coverage, there is still a probability of 0.0729 of having three or fewer unimmunized children. Thus, the decision that an area/healthcare provider is performing adequately on the basis of having three or fewer unimmunized children—of 12 children—may, in fact, be wrong in 7.29% of the time. This puts the community members at risk for they may be considered adequately covered when they are not.

Table 1 shows that, with a sample of 28 children having nine or fewer unimmunized infants in the sample, EPI centres can be classified as ‘adequately’-performing centres. Samples with more than nine unimmunized infants will be identified as ‘inadequately’-performing EPI centres. Using this rule, managers will identify areas correctly with 80% or above coverage more than 95% of the time. Similarly, they can also judge an area as inadequate if more than nine of 28 children are unimmunized in more than 95% of the time. Following these procedures, the optimum decision rules in terms of a feasible sample size and the number of uncovered allowable subjects at given levels of consumer (infants in the case of DPT1) and provider (EPI centres in the case of DPT1) risks can be formulated for various services using the binomial probabilities as was done in Table 1. The same calculation can also be done in terms of children immunized instead of unimmunized children.

While the statistical reasoning behind the LQAS method and the calculation involved may seem somewhat complicated, its application in the field is, in fact, very easy. Once the decision rules are made, ideally in consultation with policy-makers and programme managers, the task of collecting the required data and their use in deciding which of the health facilities/service providers are performing adequately or not is very simple. The method can also be used for various other outcome variables.

### Study design

The study had two components. The first component compared the performance of the LQAS method with that of the HDSS in identifying inadequately-performing work-areas. This was done by carrying out a LQAS survey and applying the LQAS decision rules to identify work-areas. The consistency between findings based on the LQAS method and the HDSS was also examined by calculating sensitivity and specificity. The second component included application of the methods by programme supervisors. This was done to assess the feasibility of training programme supervisors and the problems encountered by them in the real-life situation.

### Hypotheses

The proportion of inadequately-performing work-areas of the community health workers in the ICDDR,B-served area in Matlab as derived using the LQAS method is similar to those derived using data from the Matlab HDSS.

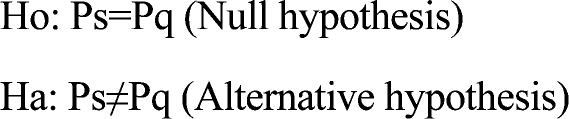


Where, Ps is the proportion of work-areas having less than L% (coverage rate below which the performance of an area will be considered inadequate) of use rates using HDSS data

and

Pq is the proportion of CHW's work-areas having less than L% of use rates derived using the LQAS technique.

### Study area

The above method was tested in Matlab, the study site of ICDDR,B. Matlab is situated approximately 55 km from Dhaka, the capital of Bangladesh. ICDDR,B has been a running a demographic surveillance system and a service-record system in Matlab for more than 25 years. The system involves monthly visits to households by female workers to collect information on births, deaths, marriage, and migration. In addition, they also provide immunization to children and family-planning supplies to couples of reproductive age. A computer-aided record-keeping system allows an accurate estimate of coverage rates for services they provide. The current population under surveillance is around 220,000. The area is divided into two with nearly half of the total population in one area. One of the areas has very intensive maternal-child health and family-planning (MCH-FP) services provided by ICDDR,B and the other half similar services by the Government of Bangladesh. The ICDDR,B services include home-delivery of fami-ly-planning supplies, EPI, distribution of oral rehydration solutions, antenatal care and postnatal care services. Fifty-seven female Community Health Workers (CHWs) provide the services at the community level. Each CHW has a defined area to cover. The LQAS method was applied to identify the inadequately-performing CHW's work-areas based on pre-defined thresholds of acceptability of DPT, measles, BCG, and contraceptive prevalence rate (CPR).

### Data collection

Data for this study came from two sources: HDSS of ICDDR,B and a cross-sectional survey following the LQAS methodology, later referred to as the LQAS survey. The latest coverage statistics by CHW's work-areas were obtained from the HDSS database. During July-September 2002, a team of trained interviewers carried out the LQAS survey. A field supervisor, a statistician, and the principal investigator of the present study supervised the fieldwork. The updated HDSS list of households with their members was used as the sampling frame.

In the LQAS survey, a systematic random sample of 30 children aged less than 24 months was selected from each CHW's work-area for assessing the inadequacy in terms of use of DPT, measles and BCG vaccinations. Children receiving any of the DPT doses were considered as having DPT. Similarly, 30 currently-married women of reproductive age, who were not pregnant at the time of the survey and with their husband living in the country, were systematically randomly selected for assessing the use of family-planning services. If any selected respondent was absent, a replacement was made from a socioeconomically similar neighbouring household.

### Data analysis

Lower and upper thresholds of service use for various indicators were decided based on current use rates of services by the target population reported by the HDSS. Table 2 shows the assumed different threshold levels for various indicators, maximum allowable individuals without any particular service to consider an area as inadequate, the associated provider, and consumer risk.

**Table 2.** Sample size, maximum allowed individuals to be adequate, and classification error

Health service	Thresholds		Sample size	Maximum no. of non-users allowed	Probability of misclassification		
	Lower (% of use)	Upper (% of use)			Provider	Consumer	Total
DPT	65	95	15	2	0.062	0.036	0.098
Measles	60	90	20	4	0.051	0.043	0.094
BCG	65	95	15	2	0.062	0.036	0.098
CPR	50	80	26	8	0.038	0.059	0.087

*CPR=Contraceptive prevalence rate

The CHW's work-areas were classified as inadequately performing using two approaches. First, using the combination of the smallest sample size and the maximum number of non-users allowed yielding the least total misclassification error (consumer and provider risk less than 0.10) as shown in Table 2. Second, the most commonly-used sample sizes, such as 19 and 28, were used with the above threshold levels of coverage to examine the level of misclassification error. Based on these two strategies, inadequately-performing CHW's work-areas were identified. At the third step, use rates of the HDSS were used for identifying inadequately-performing CHW's work-areas. CHW's work-areas with use rates lower than the lower thresholds of coverage were considered inadequately-performing areas. At the final stage, the proportion of CHW's work-areas obtained using the LQAS method and HDSS were compared for statistical significance with the null and alternative hypotheses mentioned earlier. Subsequently, classification of the work-areas by the LQAS method and HDSS was cross-tabulated to examine consistency between the two.

### Field application by supervisors

To test the feasibility of using the method by the field supervisors, the members of the project staff carried out a two-day (3–4 November 2002) training programme in Matlab. The training was attended by 19 participants, 17 of whom were from the government health and family-planning programme working in the Matlab area (not served by ICDDR,B), and the remaining participants were from Matlab health and family-planning services of ICDDR,B. Of the government programme supervisors, five were Health Inspectors, six were Assistant Health Inspectors, five Family Planning Inspectors, one EPI Technician, and two ICDDR,B Field Research Assistants. The training comprised lectures in the office and practical application in the field. A simple instruction sheet in Bangla, prepared by the project team, was given to the participants at the time of training. The training avoided technical details of the method and explained the procedures of applying the method with only sample size of 19 with varied level of thresholds. The possibility of using other sample sizes was only mentioned with an emphasis that, for most of our purposes, a sample size of 19 would be sufficient. A lot of time was spent on the method of selection of samples, replacement of absentee respondents, compilation of data, and application of the decision rule to consider an area as inadequate. The training concluded with a call to apply the method in the field as a part of their regular activities. The participants also met again after a month of applying it in the field to review and share their experiences and the problems faced in applying the method in the field.

## RESULTS

### LQAS method compared to HDSS

Table 3 presents the proportion of the inadequately-performing CHW's work-areas identified using the LQAS technique and HDSS data. For DPT, only one CHW-work area was found to be inadequate by the LQAS method compared to two when HDSS data were used. The difference in the proportions of inadequately-performing areas as identified by the two methods was not statistically significant.

**Table 3.** Proportion of health areas classified as inadequate based on LQAS (classification error less than 10%) and HDSS

Method	No. of areas	Proportion inadequate (number inadequate)			
		DPT: 65–95%	Measles: 60–90%	BCG: 65–95%	CPR: 50–80%
LQAS	57	0.02 (1)	0.09 (5)	0.02 (1)	0.05 (2)
HDSS	57	0.04 (2)	0.02 (1)	0.00 (0)	0.00 (0)
Sample size		15	20	15	26
Statistics					
Z		0.08	1.23	0.13	0.86
p		0.47	0.22	0.90	0.90

CPR=Contracptive prevalence rate;

HDSS=Health and demographic surveillance system;

LQAS=Lot quality assurance sampling

For measles, the LQAS method identified five inadequately-performing areas; while comparing with HDSS data, only one could be identified as inadequate. However, the difference in proportions was not statistically significant.

Using the LQAS technique and HDSS data, a similar consistency of identifying inadequately-performing areas was observed for contraceptive prevalence rate (CPR) and BCG. For CPR, the LQAS method identified two inadequately-performing areas compared to none by the HDSS. For BCG, only one area was identified to be inadequate by the LQAS method and none by the HDSS.

The above implied that the inadequately-performing CHW's work-areas for DPT and BCG could be identified with a sample size of 15 with less than 10% probability of misclassification. For measles and contraceptive use, a sample size of 20 and 26 respectively was enough to identify inadequately-performing areas with less than 10% misclassification error. The difference in proportion of misclassified areas identified by the LQAS method and HDSS was not statistically significant in any of the cases.

Table 4 shows the number of work-areas identified as adequate or inadequate by the LQAS method and HDSS data along with sensitivity and specificity. The table shows that, for BCG, 56 of the 57 work-areas were identified as adequate by both the systems. For DPT, 54 of the 57 work-areas were identified as adequate by both the systems. The remaining three work-areas were classified differently by the HDSS and LQAS method. For measles, 51 and, for family planning, 55 of the 57 work-areas were classified as adequate by both the systems. In all the cases, the LQAS method had a very high level of success in identifying adequately-performing work-areas and a very low level of success in identifying inadequately-performing work-areas.

**Table 4.** Consistency between HDSS and LQAS method using the schemes used in Table 3 to classify 57 work-areas

LQAS	HDSS	Adequate	Inadequate	Predictability of LQAS (%)
DPT	Adequate	54	2	Sensitivity=98.2
LQAS (n=15)	Inadequate	1	0	Specificity=0
Measles	Adequate	51	1	Sensitivity=91.1
LQAS (n=20)	Inadequate	5	0	Specificity=0
BCG	Adequate	56	0	Sensitivity=98.3
LQAS (n=15)	Inadequate	1	0	Specificity=Indeterminate
CPR	Adequate	55	0	Sensitivity=96.5
LQAS (n=26)	Inadequate	2	0	Specificity=Indeterminate

CPR=Contraceptive prevalence rate;

HDSS=Health and demographic surveillance system;

LQAS=Lot quality assurance sampling

Table 5 presents the proportion of health areas classified as inadequate with a sample size of 19. This also has identified a statistically similar proportion of inadequately-performing areas with a higher level of errors for measles and CPR. For measles, the maximum allowed number of non-immunized children—of 19 children—was four with a total misclassification error of 0.105. For CPR, the maximum allowed number of non-user couples was six to classify an area as inadequate with an error level of 0.152.

**Table 5.** Proportion of areas classified as inadequate based on 19 samples in the LQAS method and data from HDSS

Method	No. of areas	Proportion inadequate			
		DPT: 65–95%	Measles: 60–90%	BCG: 65–95%	CPR: 50–80%
LQAS	57	0.00 (0)	0.09 (5)	0.00 (0)	0.05 (3)
HDSS	57	0.04 (2)	0.02 (1)	0.00 (0)	0.00 (0)
Sample size		19	19	19	19
Statistics					
Z		0.86	1.23	Not done	1.11
p		0.39	0.22	-	0.27

Figures in parentheses indicate the number of inadequately-performing areas

CPR=Contraceptive prevalence rate;

HDSS=Health and demographic surveillance system;

LQAS=Lot quality assurance sampling

Table 6 examines the consistency between the LQAS method and the HDSS in classifying inadequately-performing work-areas and the specificity and sensitivity of the LQAS method for a sample size of 19. It can be seen from the table that there is a very high level of consistency between the two methods for the adequately-performing areas. The level of consistency for the inadequately-performing areas was also low in this case.

**Table 6.** Consistency between HDSS and LQAS method using the scheme in Table 5 to classify 57 work-areas

LQAS	HDSS	Adequate	Inadequate	Predictability of LQAS (%)
DPT	Adequate	55	2	Sensitivity=96.7
LQAS (n=19)	Inadequate	0	0	Specificity=0.0
BCG	Adequate	57	0	Sensitivity=100.0
LQAS (n=19)	Inadequate	0	0	Specificity=Indeterminate
Measles	Adequate	51	1	Sensitivity=91.1
LQAS (n=19)	Inadequate	5	0	Specificity=0.0
CPR	Adequate	54	0	Sensitivity=94.5
LQAS (n=19)	Inadequate	3	0	Specificity=Indeterminate

CPR=Contraceptive prevalence rate;

HDSS=Health and demographic surveillance system;

LQAS=Lot quality assurance sampling

Table 7 presents the proportion of the inadequately-performing areas using a sample size of 28. Here also, the proportions of the inadequately-performing areas were similar for the LQAS method and HDSS. In all the cases, the probability of misclassification was reduced.

**Table 7.** Proportion of areas classified as inadequate based on 28 samples in LQAS and use of data from HDSS

Method	No. of areas	Proportion inadequate (number inadequate)			
		DPT: 65–95%	Measles: 60–90%	BCG: 65–95%	CPR: 50–80%
LQAS	57	0.00 (0)	0.05 (3)	0.00 (0)	0.05 (3)
HDSS	57	0.04 (2)	0.02 (1)	0.00 (0)	0.00 (0)
Sample size		28	28	28	28
Statistics					
Z		0.86	0.36	Not done	1.11
p		0.39	0.72	-	0.27

CPR=Contraceptive prevalence rate;

HDSS=Health and demographic surveillance system;

LQAS=Lot quality assurance sampling

The consistency between the HDSS and the LQAS results are presented in Table 8. The table shows that all the work-areas were considered adequate by both HDSS and LQAS method for BCG, 55 for DPT, 53 for measles, and 54 for family planning.

**Table 8.** Consistency between HDSS and LQAS method using the scheme in Table 7 to classify 57 work-areas

LQAS	HDSS	Adequate	Inadequate	Predictability of LQAS (%)
DPT	Adequate	55	2	Sensitivity=100.0
LQAS (n=28)	Inadequate	0	0	Specificity=0.0
Measles	Adequate	53	1	Sensitivity=94.6
LQAS (n=28)	Inadequate	3	0	Specificity=0.0
BCG	Adequate	57	0	Sensitivity=100.0
LQAS (n=28)	Inadequate	0	0	Specificity=Indeterminate
CPR	Adequate	54	0	Sensitivity=94.7
LQAS (n=28)	Inadequate	3	0	Specificity=Indeterminate

CPR=Contraceptive prevalence rate;

HDSS=Health and demographic surveillance system;

LQAS=Lot quality assurance sampling

### Feasibility of using LQAS by field-level supervisors

It was revealed that, both during training and application of the LQAS method that most supervisors could understand the procedures to be adopted in making the decision and conducting the survey. Use of the register of clients they maintain for services as a sampling frame was also convenient for them.

In the review session after a month, it was revealed that two of the 17 supervisors did not use LQAS due to shortage of time. Those who did use it reported the following problems: (a) lack of accurate information on the number of households in their work-area, (b) selection of respondents for collecting data on various service components, and (c) decision about the lower and upper threshold levels of service coverage for identifying inadequately-performing areas.

## DISCUSSION

While the proportion of areas in terms of use of health services based on the LQAS method was statistically similar to those obtained using HDSS data, the LQAS method seemed to perform better in our case in identifying the adequately-performing areas than in identifying the inadequately-performing areas. This could be due to the peculiarity of the area with a very high coverage of services, leaving a very small number of the inadequately-performing areas. This observed poor performance of the LQAS method in identifying the inadequately-performing areas should not be of concern for the magnitude of the probability of misclassification should remain within the limit set under the LQAS scheme. It should, however, be mentioned that, although the theoretical basis of the LQAS method is sound, its performance in settings with medium and low coverage cannot be concluded based on this study. It is reported, however, that the method is not very useful in areas with less than 20% coverage (11).

One of the real challenges is to adopt the LQAS method in the regular monitoring system at the lowest level of the programme. It is clear that the theoretical basis of the LQAS method will be too complicated for supervisors. A practical difficulty in its use by programme supervisors was the computation of the sample size corresponding to the different threshold levels of coverage and their associated level of error. One way of simplifying the task is to seek for a fixed sample size, as small as possible, which is reasonably good in terms of level of error for different threshold levels. If one examines the total classification error in Table 1, it is apparent that the sample sizes with the lowest level of error were 28 and 19. This points to the possibility of whether 19 could be used as a sample size for all levels of threshold points and what implications it would have on the magnitude of misclassification. Table 9 presents the maximum number of non-users allowed—of 19—with associated magnitude of errors under various levels of thresholds.

**Table 9.** Magnitude of classification error with various upper and lower threshold levels with sample size of 19 in the LQAS method

Parameter	Threshold levels (lower-upper)											
	10–40	15–45	20–50	25–55	30–60	35–65	40–70	45–75	50–80	55–85	60–90	65–95
Maximum no. of non-users allowed	14	13	12	11	10	9	8	7	6	5	4	3
Consumer risk	0.070	0.078	0.084	0.087	0.088	0.087	0.084	0.077	0.068	0.054	0.035	0.013
Provider risk	0.035	0.054	0.068	0.077	0.084	0.087	0.088	0.087	0.084	0.078	0.070	0.059
Total risk	0.105	0.132	0.152	0.164	0.172	0.174	0.172	0.164	0.152	0.132	0.105	0.072

It can be seen from Table 9 that if one is ready to accept the level of consumer and provider risk less than 10% each than a sample size of 19 will serve the purpose for varied levels of threshold points. In such circumstances, the degree of misclassification of a work-area will be slightly higher, implying that, in the long run, one will misclassify 20 work-areas instead of 10 per 100. But to bring down the error level to less than 10%, the sample size may need to be increased up to 28 from 19, and in real-life resources needed to survey 28, instead of 19, are much higher. The trade-off is to be happy with a slightly higher level of error and reduced costs for the survey. Thus, for many practical purposes, a sample size of 19 should serve the purpose for programme managers.

At the field level, lack of a up-to-date sampling frame can pose a challenge to applying the method. In such circumstances, emphasis should be placed on updating the list as regularly as practical which not only makes the method easily adoptable but also may serve other programmatic purposes. If there is no sampling frame, it may be appropriate to adopt other methods for the selection of a simple random sample, such as going to the centre of a village, then spinning a bottle, or a similar object to select a direction to move to select the required number of respondents in a manner by ensuring spread over the village in that direction. The LQAS method assumed that the samples are selected at random from a list of respondents; so, the use of cluster-sampling scheme in selecting samples will require an upward adjustment of the sample size; the magnitude of adjustment will, however, depend on the size of intra-class correlation within clusters.

## ACKNOWLEDGEMENTS

The study was supported by USAID/Dhaka through its targeted research grant to ICDDR,B. The authors are also grateful to Mr. A.Z. Khan, Field Research Officer at the Social and Behavioural Sciences Unit, ICDDR,B, for his assistance in carrying out the field work.
